# A fast and high precision multi-robot environment modeling based on M-BFSI: Bidirectional filtering and scene identification method

**DOI:** 10.1016/j.isci.2024.109721

**Published:** 2024-04-10

**Authors:** Dai-ming Liu, Jia-shan Cui, Yong-jian Zhong, Chang-wan Min, Fang-rui Zhang, Dong-zhu Feng

**Affiliations:** 1 School of Aerospace Science and Technology, Xidian University, Xi’an 710126, China; 2 Shanghai Electro-Mechanical Engineering Institute, Shanghai 201109, China; 3 China Academy of Space Technology, Xian Branch, Xi’an 710100, China

**Keywords:** Control engineering, Robotics, Automation

## Abstract

This article designs and implements a fast and high-precision multi-robot environment modeling method based on bidirectional filtering and scene identification. To solve the problem of feature tracking failure caused by large angle rotation, a bidirectional filtering mechanism is introduced to improve the error-matching elimination algorithm. A global key frame database for multiple robots is proposed based on a pretraining dictionary to convert images into a bag of words vectors. The images captured by different sub-robots are compared with the database for similarity score calculation, so as to realize fast identification and search of similar scenes. The coordinate transformation from local map to global map and the cooperative SLAM exploration of multiple robots is completed by the best matching image and the transformation matrix. The experimental results show that the proposed algorithm can effectively close the predicted trajectory of the sub-robot, thus achieving high-precision collaborative environment modeling.

## Highlights

• A two-way screening mechanism is introduced to improve the error-matching elimination algorithm
• Build a keyframe database to identify and search of similar scenes

## Graphical abstract

**Figure:**
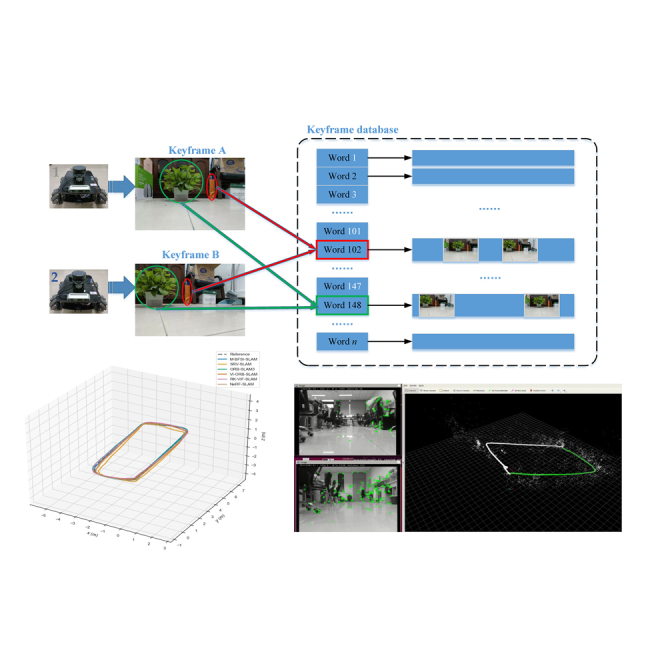

## Introduction

With the rapid development of modern science and technology, robots with different forms are widely used in military operations, science, education and research, disaster prevention and control, and other fields.^1^^,^^2^^,^^3^^,^^4^ For example, in deep space exploration, robots are used to carry out early exploration in specific areas, so as to for-mulate more in-depth scientific research plans; A variety of robots are used in disaster prevention to help humans perform dangerous tasks. With the development of science and technology and the increasing demand for detection equipment, more high-performance equipment is also used in the work. Before using robots to carry out research on an unknown environment, it is urgent to use robots to carry sensors to conduct intuitive and accurate scene modeling of the exploration environment,^5^^,^^6^ so as to provide great convenience for the implementation of subsequent tasks.

At present, although robots have been successfully used in real scenes, for large working environments or relatively complex production operations, such as automatic transportation robots sorting materials in large warehouses, or automatic explosive removal robots carrying out large-scale explosive removal operations, it will be obviously inadequate to rely on one automatic robot to complete a variety of complex tasks. It is mainly reflected in the low work efficiency and low fault tolerance of robot system. Inspired by the division of labor and cooperation in human society, some scholars turned their attention to the cooperative work of multiple robots. Therefore, when faced with more complex large-scale production and operation situations, the viewpoint that multiple automatic robots are arranged at the same time to assist in jointly completing various tasks is proposed. Through this method, more complex tasks can be completed. at the same time, the robustness, scalability and other advantages of multi-robot system also make it more widely used than single-robot system.^7^^,^^8^^,^^9^^,^^10^ At present, the re-search on multi-robot cooperative SLAM mainly focuses on multi-robot information association, multi-robot map mosaic, and overall position and attitude optimization.

One of the key problems of multi-robot cooperative SLAM is how to establish the relationship between robot members. Scholars have done a lot of research on this issue.^11^^,^^12^^,^^13^ With the development of visual image, digital image processing has opened up many research directions, such as target detection and tracking. How to combine visual image information to establish information association between machines has become a concern of some scholars. Howard^14^ proposed the Gaussian-based Probabilistic Map Merging (PMM) in 2016. This method is implemented on the premise that the initial position and pose of the sub-robots are known, or the sub-robots can meet in the line of sight, and multiple sub-maps can be fused by combining the relative transformation matrix between the robots and the Map Merging Bases (MMBs). The author has proved the effectiveness and fusion accuracy of the proposed algorithm through hardware experiments, but its disadvantage lies in the use of particle filtering in the algorithm, resulting in strong dependence on particle number and particle diversity. Literature^15^ proposed a vision SLAM collaboration framework for multiple robots. The framework executes LSD-SLAM^16^ separately on each sub-robot, predicts seven state quantities of the camera in rotation, position and scale, and establishes a sub-map about itself. The central processing unit is responsible for judging whether the sub-robots have reached the common area. If there is a common area, the transformation matrix is calculated to fuse the sub-maps, and finally establish an overall consistent sparse map form. At present, this method has become a template for subsequent multi-robot collaborative SLAM scheme design. Literature^17^ proposed a multi-robot SLAM scheme based on path graph optimization. This method constructs a path graph consisting of only nodes and edges for each sub-robot. The node includes two aspects, namely, the predicted position and pose value and the map point under the position and pose; The edge is the transformation matrix obtained by feature matching between nodes. After the recognition of multiple sub-robots in the field of vision is successful, the data required for map fusion is generated through information exchange. When all sub-robots complete the detection task, all sub-maps are uploaded to the server, and the server will fuse different sub-maps through the map fusion data returned.

For the problem of map mosaic of multiple robots, the data types generated by different sensors carried by robots are different, which leads to different map representations of the environment. The map forms constructed according to SLAM tasks can be divided into occupancy grid map and point cloud map. Among them, the most pioneering method in the field of 3D point cloud map mosaic is iterative closest point (ICP).^18^^,^^19^ Its disadvantage is that the algorithm needs good initialization, otherwise it will fall into local optimization. For the defects of ICP, after years of development, re-searchers have proposed a variety of variants of ICP algorithm,^20^ such as PL-ICP,^21^ NICP,^22^ IMLS-ICP,^23^ etc. In addition to ICP and its variant algorithms, some researchers^24^ realized the splicing of multi-robot SLAM sub-maps to global maps with the idea of adaptive Monte Carlo localization (AMCL). The algorithm executes Fast SLAM on each sub-robot, and the sub-robot construction map includes map point observation and self-position estimation. In the process of map mosaic, AMCL is used to locate all other sub-robots in a sub-robot map, and map overlapping information is used to complete the mosaic of multiple sub-maps. 3D point cloud can well represent the contour features of the detection scene, but it cannot be directly used for navigation tasks. For this reason, Jessup^25^ and others first proposed to use Octree formed based on 3D point cloud for map mosaic in 2015. Compared with the original 3D point cloud map, Octree not only has the advantages of high map resolution, but also can express a larger scene under the same memory conditions. It has been proved that the system can be effectively transplanted to the SLAM scheme of mobile multi-robots. As early as in the single-robot SLAM, a large number of scholars have proposed different excellent solutions based on the idea of graph optimization.^26^^,^^27^^,^^28^ Expanding to the field of multi-robot SLAM, Kim^29^ proposed a multi-robot collaborative positioning and mapping algorithm. The algorithm absorbs the idea that single-robot SLAM uses incremental smoothing and mapping (ISAM)^30^ to solve nonlinear optimization problems in real-time, and improves it and applies it to multi-robot SLAM. The sub-ma-chines realize “indirect encounter” on the premise that the same road punctuation points are observed at any time, and complete the transformation matrix calculation based on this, and then update the state quantity of the global map using the ISAM method. The authors use a variety of heterogeneous robots to experiment, and verify the effectiveness of the proposed algorithm in different scenarios. Lazaro^31^ proposed a multi-graph optimization SLAM method for compressing measurement information. This method executes visual odometer to build sub-map before sub-robots meet, and only needs to exchange compressed measurement data after meeting. This approach greatly reduces the amount of data to be processed when optimizing the back-end graph of the algorithm, reduces the transmission burden of the communication module, and enhances the overall robustness of the multi-machine system. Indelman^32^ proposed a multi-robot collaborative positioning method based on global pose map optimization. This method does not need to obtain the initial relative position and attitude of the robot in advance, and the local to global transformation relationship is solved through part of the common observation area. At the same time, in order to avoid the matching error between different sub-robots, the expectation maximization (EM) probability method is used to infer the initial position and attitude of the sub-robots and solve the data association problem, so as to improve the overall map accuracy and robustness to abnormal value interference. Stuart^33^ and others developed a multi-camera collaborative 3D reconstruction system. The author improves the optimization algorithm of the rear-end pose map, which makes the system can effectively eliminate the error drift between image frames, thus greatly reducing the probability of error matching between maps created by different robots. The improved algorithm effectively avoids the problem of scene change caused by too long reconstruction time, and realizes large-scale scene 3D reconstruction with good global consistency.

To sum up, a large number of scholars have deeply explored the problems in multi-robot SLAM and proposed many excellent solutions. Some researchers focus on exploring the multi-robot system architecture, and some focus on analyzing the information association and interaction mode between robots. Some scholars tried to improve the map mosaic method by combining existing algorithms, and some researchers explored a globally consistent optimization method for outliers in the map. Although some achievements have been made in these researches, up to now, the cooperative mapping algorithms of multi-robot systems with excellent results are still rare, and most of them require high computing power and high-cost hardware resources to support. This article is based on the vision-based single-robot SLAM algorithm. In order to solve the problem of feature tracking failure caused by large angle rotation during robot exploration, a two-way screening mechanism is introduced to improve the error-matching elimination algorithm, which improves the robustness of the algorithm while retaining the correct feature matching. Then, using the idea of pretraining dictionary to convert images into a bag of words vectors, it is proposed to build a global key frame database of multiple robots, realize fast identification and search of similar scenes, complete effective sub-map building and global map splicing for multi-robot systems, and provide an efficient and high-precision solution for multi-robot collaborative mapping.

## Multi-robot cooperative simultaneous localization and mapping system framework

**Figure 1:**
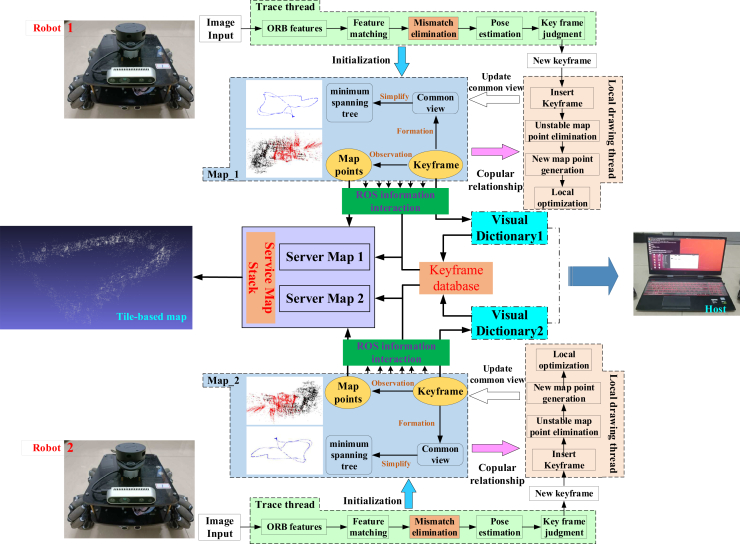
Multi-robot system framework

The system framework of the multi-robot collaborative SLAM method proposed in this article is shown in Figure 1. It includes two clients and a central server that can communicate with clients. The communication relationship between the two parties is established through the local area network. Each client is configured with a binocular camera, and the system does not assume any prior knowledge about the client. Each client runs the visual front-end independently, and its reference coordinate system origin is located at its own initial exploration position.

**Figure 2:**
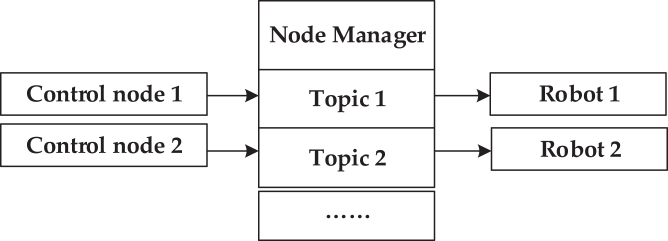
Information interaction framework

In the multi-robot cooperative system, the communication channel of robots is established through information interaction, so that the collaborative SLAM task designed in this article can be completed without mutual knowledge. As shown in Figure 2, node information interaction is completed through the node manager.

To meet the requirements of this article, a key frame database of multiple robots is built and stored with a laptop as the server. At the same time, take a single mobile robot as the client, and execute the visual front-end respectively. The single-robot packs the maintained camera pose and local map information into a bag file, and sends a message package for the server to subscribe. The server identifies similar scenes through the key frame database built in real-time, so as to establish the relationship between camera positions and map points among multiple machines.

## Single-robot vision simultaneous localization and mapping method based on bidirectional filtering

In order to make full use of computing resources, the vision front-end framework of single-robot adopts the idea of double threads, which includes two parts: tracking thread and local mapping thread (as shown in Figure 1). The basic functions of the two threads are as follows:

Tracking thread: the thread input data is a single frame image, and its functions mainly include:

(1) Extract feature points from image frames input into the system, and conduct feature matching and error-matching elimination. Use correct matching point pairs to estimate camera motion between images; At the same time, we use the binocular ranging principle to calculate the depth data of matched feature points and convert them into 3D map points;
(2) Judge whether the frame meets the conditions for becoming a key frame. If it meets the conditions, the frame will be converted into a key frame and ready for output to subsequent threads.

Local mapping thread: the input of this thread is the filtered key frame output by the previous thread. Its main function is to maintain a certain number of keyframes and express the surrounding environment with the highest efficiency. The specific calculation steps of this thread are as follows:

(1) Update the common view and minimum spanning tree after inserting keyframes, and add 3D map points recovered from correctly matched features;
(2) Select the map point generated by three consecutive frames of inspection in the common view. If there is a map point that cannot be observed by three frames at the same time, it is considered that the map point is unstable, and it will be deleted;
(3) In order to ensure the dynamic balance of the number of local map points, it is necessary to restore some new map points. In order to insert 2D features of map points that have not been mapped in the key frame, find the correct matching relationship for them in the history frame and restore their 3D coordinates. If the recovered map point can be observed by three adjacent frames, it indicates that the map point is in a stable state and is a new map point;
(4) Take the pose estimation obtained from the previous thread as the initial value, and use the bundle adjustment (BA) to optimize all the pose and map point coordinates in the local map;
(5) Filter and filter out redundant keyframes, and complete the optimization.

The map is composed of key frames, map points, common views, and minimum spanning trees. The details are as follows:

(a) Key frame: extracted features and their descriptors, word bag vector of left eye image and camera pose;
(b) Map points: world coordinates of feature points;
(c) Common view: it is composed of points and multiple edges connecting each point. Points represent camera positions, edges represent the same map points observed by two cameras, and the weight of edges is the number of the same map points;
(d) Minimum spanning tree: the smallest subset of the common view. All keyframes (nodes) are retained, and every two nodes are connected only by the edge with the highest weight. The common view is simplified to the greatest extent, which is convenient for closed-loop detection and calculation.

Aiming at the mismatch problem caused by large angle rotation, this article pro-poses an improved two-way filtering RANSAC method. Before using the mismatch elimination method, two rounds of rough elimination are performed on the matching point pairs with errors. By improving the initial value of the RANSAC algorithm input, increasing the number of correct matching points, and reducing the uncertainty of the original RANSAC algorithm. The algorithm inputs a given pair of matching points with errors, and records the two point sets as $X=\left\{x_{1},x_{2},...,x_{n}\right\}$ and $y=\left\{y_{1},y_{2},...,y_{n}\right\}$ . Select four pairs of points with the best quality to calculate the homography matrix *H*. Specifically, this article uses the ratio test method to assign a confidence score to each matching point pair. That is, for matching frames $KF_{A}$ and $KF_{B}$, take a feature point in $KF_{A}$ and find the first two features that are closest to the Hamming distance of the descriptor in image $KF_{B}$. Record the closest distance as *distance*_*m1*_, and the next closest distance as *distance*_*m2*_. The ratio μ is used as the confidence score for the point pair:

(Equation 1) $$\mu =\frac{{distance}_{m1}}{{distance}_{m2}}$$

Wherein, the smaller the value of μ, the greater the probability that the point pair is the correct matching point pair. Select the four sets of points with the lowest μ value and calculate the homography matrix *H* between the two point sets. Using the idea of bidirectional filtering, for any point $x_{i}$ in the point set *X*, the corresponding point $y_{x_{i}}$ in the point set *Y* is calculated using the matrix; Similarly, for any point $y_{i}$ in point set *Y*, its corresponding point $x_{y_{i}}$ in point set *X* is calculated. Given the threshold value Δδ, if the pixel distances between point $x_{i}$ and point $x_{y_{i}}$, and between point $y_{i}$ and point $y_{x_{i}}$ are both less than Δδ, then point $x_{i}$ and point $y_{i}$ are considered as a set of matching points, otherwise these two points are directly removed from the origin set. Since then, the first round of rough culling results has been obtained.

**Figure 3:**
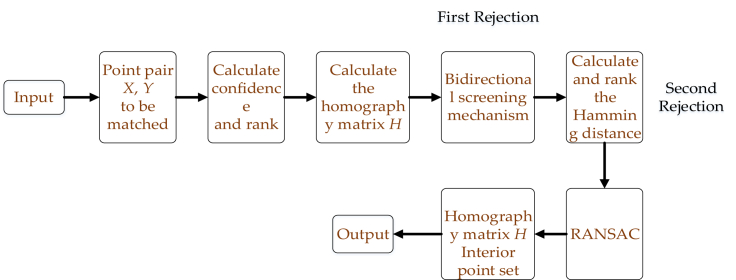
Flow chart of feature mismatch rejection

After the first round of rough elimination, a large number of point pairs with particularly obvious errors were eliminated. However, only four sets of points are used in the calculation of the homography matrix *H*. At this time, the *H* matrix does not necessarily describe the optimal correspondence between the two-point sets, so there may still be some mismatched point pairs that are difficult to be eliminated by the bi-directional filtering mechanism. To solve this problem, this article proposes a second round of rough matching, which is to calculate and sort the Hamming distance of the selected matching point pairs, select the first 10% point pairs with the largest Hamming distance value, and eliminate them as erroneous matching points. After two rounds of rough matching, the results are inputted into the RANSAC algorithm for the iterative solution. The method proposed in this article effectively improves the initial input value of the RANSAC algorithm, while retaining more correct feature point pairs, reducing the algorithm uncertainty caused by input errors. The algorithm flow is shown in Figure 3.

## Map Splicing Strategy

Section single-robot vision simultaneous localization and mapping method based on bidirectional filtering of this article describes the SLAM method used in a single-robot system. In scenarios of the same size, multi-robot collaboration often has higher work efficiency, which is the original intention of this article to expand to multi-robot collaborative SLAM schemes based on the single-robot SLAM scheme. The research focuses on multi-robot collaboration covers issues such as information interaction between robots, sub-robot map construction, and sub-map stitching. Section Multi-robot cooperative simultaneous localization and mapping system framework introduces the information interaction scheme used between multiple robots. This section solves the most critical problem of submap splicing.

### Building keyframe database

After completing the construction of the scene dictionary, use the dictionary to convert the image into a numerical vector. TF-IDF is used to calculate the weights $\eta_{w1}$, $\eta_{w2}$, $\eta_{w3}$ …, of each word in the image, and ultimately generate a description vector $V_{p}$:

(Equation 2) $$V_{p}=\left[\begin{array}{cccc} \eta_{w1} & \eta_{w2} & \cdots & \eta_{wn} \end{array}\right]$$

**Figure 4:**
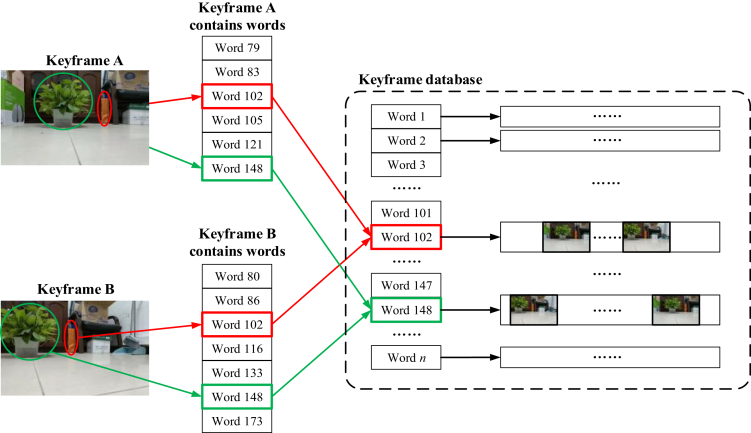
Key frame database

After obtaining the description vector for each frame, a key frame database is incrementally constructed during the operation of the multi-robot system, which contains all key frames obtained from all sub-robots. For subsequent similar scene recognition tasks, this article adds numbering information when calculating the description vector of a key frame. The numbering content includes the numbering value of the sub-robot it belongs to in the team. The size of the training dictionary is taken as the key frame database capacity. For each type of word, there are key frames that form an inclusion relationship with them. Therefore, the format of the key frame database is shown in Figure 4 below. All words form a primary directory, with a linked list corresponding to each word. The linked list stores the key frame that owns the word.

When searching the key frame database, since the words of the current frame are known, using anti-indexing can efficiently find key frames with the same characteristics from historical frames. When deleting a key frame, because each frame contains multiple words, first traverse the word vector corresponding to the frame, and then traverse the key frame list in the words contained in the frame to delete the same key frames under the list.

### Identification of similar scenarios

**Figure 5:**
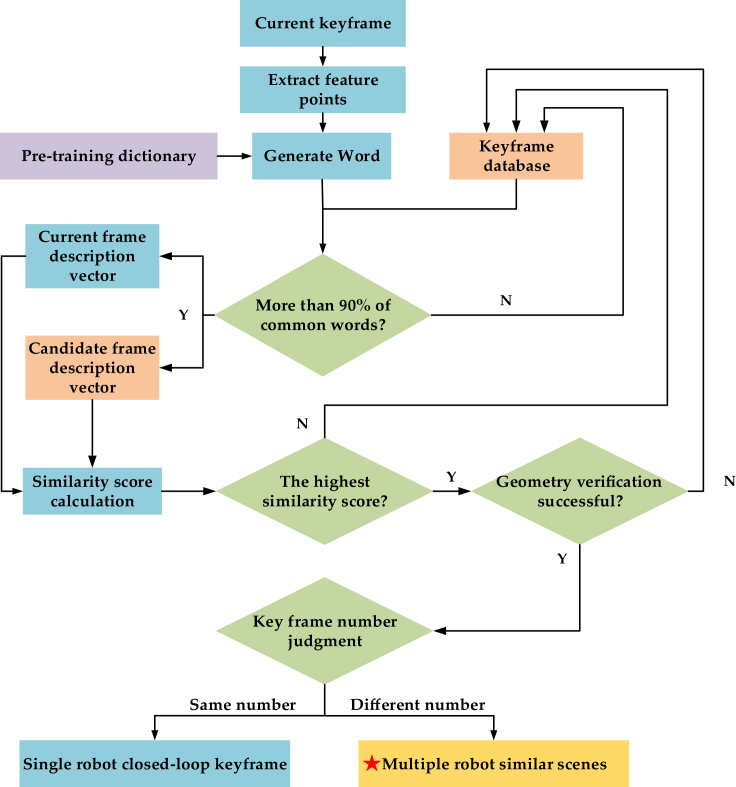
Key frame database

The key frame database constructed in the previous section is used to identify similar scenes and calculate the positional transformation relationship between them, thereby completing the point cloud map mosaic. Figure 5 shows the process of identifying similar scenarios.

In similar scene recognition, first extract ORB features from the new key frame, and then use the trained scene dictionary to generate a description vector for the current image frame. In order to prevent mis-identification, it is necessary to set strict filtering conditions, that is, the number of common words between the current frame and the historical frame exceeds 90%. Only when this condition is met can we proceed to the next step for similarity score calculation. Otherwise, the current frame will be added to the key frame database. Note that the current frame is $KF_{A}$ and the candidate historical frame is $KF_{B}$, and their image description vectors are $V_{A}$ and $V_{B}$, respectively. The similarity score $S\left(V_{A}-V_{B}\right)$ is calculated using the $L_{1}$-norm:

(Equation 3) $$S\left(V_{A}-V_{B}\right)=1-\frac{1}{2}\left|\frac{V_{A}}{\left|V_{A}\right|}-\frac{V_{B}}{\left|V_{B}\right|}\right|$$

Traverse the entire database to obtain the highest similarity score. So far, the appearance consistency verification for similar scenarios has been completed. In order to further constrain the accuracy of identification, geometric consistency verification is also required, which divides the features matched to the current frame and candidate frame into inner points and outer points based on the error size. The matching frame pair is considered correct only when the number of interior points reaches the threshold.

After verifying the appearance and geometric consistency, the last step of the scene recognition module is to determine the number information of the current frame and the candidate frame. If the current frame and the candidate frame come from the same robot and the difference between the two frames exceeds 10 frames, the local map closed-loop optimization of the robot is performed within the service map stack using the current frame as the closed-loop point; On the contrary, it indicates that the two frames come from different robots, and the matching key frame pair at this time is a similar scene, which is used as the input for the next relative pose calculation.

### Calculation of relative posture of robot members

#### Robot coordinate system

**Figure 6:**
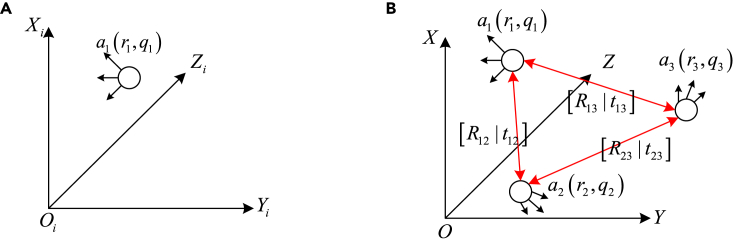
Robot coordinate system (A) single-robot coordinate system; (B) multi-robot coordinate system.

Within the entire multi-robot system, each robot takes its initial exploration time as the local coordinate system origin. For multi-robot systems, it is necessary to determine a global coordinate system. In this article, the local coordinate system of robot 1 is used as the global coordinate system. As shown in Figure 6 (a) shows the motion state of a single robot itself at a certain moment. (b) shows the kinematic relationship between multiple robots. Camera pose (r,q) consists of two parts: three-dimensional coordinates and quaternions. $\left[R_{ij}|t_{ij}\right]$ is the positional transformation relationship from robot i to robot j.

#### Calculation of relative posture of robots

Through image similarity calculation through the key frame database, a group of images that meet the conditions has been obtained. By performing a series of operations on this group of images, such as extracting features, matching features, and eliminating mismatches, multiple sets of successfully matched feature point pairs can be obtained. Using the corresponding relationship between two-dimensional image points, it is possible to restore the relative posture between two frames of images, that is, the relative posture between machines.

Note that the two matching image frames are respectively $KF_{1}$ and $KF_{2}$, and assume that the pose relationship between the images is T=[R|t]. For any spatial point P observed jointly by the two frames, assume that its projection points on $KF_{1}$ and $KF_{2}$ are $p_{1}$ and $p_{2}$, respectively. Using the camera’s aperture imaging model, the following homogeneous relationship is obtained:

(Equation 4) $$\left\{ \begin{array}{l} p_{1}=KP\\ p_{2}=K\left(RP+t\right) \end{array}\right.$$

Where K is the camera internal reference. Taking $x_{1}=K^{-1}p_{1}$ and $x_{2}=K^{-1}p_{2}$, the above equation becomes:

(Equation 5) $$x_{2}=Rx_{1}+t$$

Do the outer product with t at both ends of the above equation, and record it as $t^{\wedge }$.

(Equation 6) $$t^{\wedge }x_{2}=t^{\wedge }Rx_{1}$$

Then multiply both sides of the equation by $x_{2}^{T}$：

(Equation 7) $$x_{2}^{T}t^{\wedge }x_{2}=x_{2}^{T}t^{\wedge }Rx_{1}$$

Since the left end $t^{\wedge }x_{2}$ of the equation is perpendicular to both t and $x_{2}$, the left end $x_{2}^{T}t^{\wedge }x_{2}=0$ of the equation is obtained as follows:

(Equation 8) $$x_{2}^{T}t^{\wedge }Rx_{1}=0$$

The expression is called a polar constraint, and its geometric meaning is that the spatial point P, the projection points $p_{1}$ and $p_{2}$ are coplanar. The above equation contains both rotation and translation information. The matrix $E=t^{\wedge }R$ is called the essential matrix. Therefore, the inter frame pose estimation problem is converted into the following two sub-problems:

(1) Calculate the matrix E based on the pixel coordinates of the matching point pair;
(2) Solve R and t using matrix E.

For sub-problem 1, consider a pair of matching points first. Note that the normalized coordinates are $x_{1}={\left[u_{1},v_{1},1\right]}^{T}$, $x_{2}={\left[u_{2},v_{2},1\right]}^{T}$, and E are a 3 × 3 matrix. Write the equation as follows:

(Equation 9) $$\left[\begin{array}{ccc} u_{1} & v_{1} & 1 \end{array}\right]\left[\begin{array}{ccc} e_{11} & e_{12} & e_{13}\\ e_{21} & e_{22} & e_{23}\\ e_{31} & e_{32} & e_{33} \end{array}\right]\left[\begin{array}{c} u_{2}\\ v_{2}\\ 1 \end{array}\right]=0$$

Expand matrix:

(Equation 10) $$\left[\begin{array}{ccccccccc} u_{1}u_{2} & u_{2}v_{1} & u_{2} & u_{1}v_{2} & v_{1}v_{2} & v_{2} & u_{1} & v_{1} & 1 \end{array}\right]\left[\begin{array}{c} e_{11}\\ e_{21}\\ e_{31}\\ e_{12}\\ e_{22}\\ e_{32}\\ e_{13}\\ e_{23}\\ e_{33} \end{array}\right]=0$$

The same relationship exists for other matching point pairs. In the formula, because the antipolar constraint is a constraint with an equality of zero, and the constraint relationship for matrix multiplication E is still satisfied with any non-zero constant, it is said that E has scale equivalence. Considering that E has 9 unknown variables and the matrix has scale invariance, take the matched 8 pairs of points and form a linear equation system in the form of the equation:

(Equation 11) $$\left[\begin{array}{ccccccccc} u_{1}^{1}u_{2}^{1} & u_{2}^{1}v_{1}^{1} & u_{2}^{1} & u_{1}^{1}v_{2}^{1} & v_{1}^{1}v_{2}^{1} & v_{2}^{1} & u_{1}^{1} & v_{1}^{1} & 1\\ u_{1}^{2}u_{2}^{2} & u_{2}^{2}v_{1}^{2} & u_{2}^{2} & u_{1}^{2}v_{2}^{2} & v_{1}^{2}v_{2}^{2} & v_{2}^{2} & u_{1}^{2} & v_{1}^{2} & 1\\ \vdots & \vdots & \vdots & \vdots & \vdots & \vdots & \vdots & \vdots & \vdots \\ u_{1}^{8}u_{2}^{8} & u_{2}^{8}v_{1}^{8} & u_{2}^{8} & u_{1}^{8}v_{2}^{8} & v_{1}^{8}v_{2}^{8} & v_{2}^{8} & u_{1}^{8} & v_{1}^{8} & 1 \end{array}\right]\left[\begin{array}{c} e_{11}\\ e_{21}\\ e_{31}\\ e_{12}\\ e_{22}\\ e_{32}\\ e_{13}\\ e_{23}\\ e_{33} \end{array}\right]=0$$

Wherein, the superscript on the right represents the first i point. The matrix E can be obtained by solving the linear equations. Sub-problem 1 solved.

For sub-problem 2, this article uses singular value decomposition to recover the process of R and t from E. The singular value decomposition of matrix E is as follows:

(Equation 12) $$E=U\sum V^{T}$$

Where U and V are orthogonal matrices, and ∑ is a singular value matrix. There are two possible R and t correspondences for any E:

(Equation 13) $$\begin{array}{l} t_{1}^{\wedge }=UR_{Z}\left(90^{\circ }\right)\sum U^{T}，R_{1}=UR_{Z}^{T}\left(90^{\circ }\right)V^{T}\\ t_{2}^{\wedge }=UR_{Z}\left(-90^{\circ }\right)\sum U^{T}，R_{2}=UR_{Z}^{T}\left(-90^{\circ }\right)V^{T} \end{array}$$

Wherein, $R_{Z}\left(90^{\circ }\right)$ represents rotating R by 90° along the Z axis, and $R_{Z}\left(90^{\circ }\right)$ is the same. Therefore, there are four sets of possible solutions from E decomposition to R and t. There are implicit constraints in the camera projection model, that is, the depth values of the projection points are all positive. Substituting these four sets of solutions into matching point pairs to calculate coordinates can obtain a unique set of solutions that make the depth value positive. This sub-problem 2 is solved.

By decomposing the problem of inter frame pose estimation, the relative pose matrix T=[R|t] of two frames under 8 pairs of matching points is obtained. However, other matching point pairs in the two frames of images have not been optimized, which may lead to the displacement of the spliced map, resulting in an increase in map redundancy. Therefore, based on this, using the obtained matrix *T* as the initial value, a global BA optimization is performed for the matching point pairs on the entire image. Add the depth information of the spatial point P on the projection frames $KF_{1}$ and $KF_{2}$ based on the Equation 4, including:

(Equation 14) $$\left\{ \begin{array}{l} s_{1}p_{1}=KP\\ s_{2}p_{2}=K\left(RP+t\right) \end{array}\right.$$

Note that the number of feature point pairs successfully matched on two frames of images is N, and construct the least square problem:

(Equation 15) $$\arg\min_{R,t,P}\sum_{i=1}^{N}\left({\left\|s_{1}^{i}p_{1}^{i}-KP^{i}\right\|}^{2}+{\left\|s_{2}^{i}p_{2}^{i}-K\left(RP^{i}+t\right)\right\|}^{2}\right)$$

The superscript in the formula represents the *i*-th point. The iterative results of the Gaussian Newton (G-N) method can be obtained:

(Equation 16) $$J^{T}J\Delta x=-J^{T}\varepsilon$$

Where J represents the Jacobi of all variables in the equation, and Δx represents the increment to be solved. During iterative updating, a descent factor λ is added to constrain the error change direction:

(Equation 17) $$\left(J^{T}J+\lambda diag\left(J^{T}J\right)\right)\Delta x=-J^{T}\varepsilon$$

In which, adjust λ so that the error change always toward the decrease of ε. When solving the equations, combined with the sparse structure of the coefficient matrix $J^{T}J+\lambda diag\left(J^{T}J\right)$, the Schur elimination idea can be used to reduce the matrix dimension and accelerate the calculation, thereby solving the optimal pose transformation $T^{*}$ optimized by the beam method adjustment.

### Map splicing

We take Robot 1 and Robot 2 as the specific analysis objects. According to the definition, Robot 1 is a team coordinate system. The sub-map of Robot 2 is converted into a team coordinate system through the posture matrix $T^{*}$. The expression for the map points and key frame posture stitching process is shown below.

(Equation 18) Pi′2=[R∗t∗01]·Pi2,i=1,2,3,⋯,M

(Equation 19) Tj′2=[R∗t∗01]·Tj2,j=1,2,3,⋯,N

In Equation 18, Pi2 and Pi′2 respectively represent the map points of Robot 2 before and after conversion, and M represents the total number of map points in Robot 2; In Equation 19, Tj2 and Tj′2 represent the key frame positions and poses of Robot 2 before and after conversion, respectively, and N represents the total number of key frame positions and poses of Robot 2.

After the above transformation, a true global map has not yet been formed. Robot 1 and Robot 2 complete similar scene detection at a certain moment, marking the moment of Robot 1 as k. Therefore, the resulting map is in the k-time coordinate system. Transfer it to the initial coordinate system (k=0) of Robot 1 to complete the creation of the global map.

(Equation 20) Pi′1=(T01·T11·…·Tk1)·Pi1,i=1,2,3,⋯,M+Q

Wherein, T01, T11 and Tk1 represent the respective posture matrices of robot 1 from the initial moment to the k-time, and M+Q represents the total number of map points after splicing.

## Simulation experiment and result analysis

### Visual feature point extraction and matching experiment

In order to verify the feasibility of the proposed error-matching elimination method based on the bidirectional filtering strategy (BFS), this article compares and analyzes the improved error-matching elimination method with the RANSAC method. Aiming at the problem of positioning failure caused by too few matching points between frames due to the large angle rotation of a mobile robot in a real scene, this article simulates a large angle rotation scene using 30°, 40°, 50°, 60°, 70°, and 80° as the shooting angle difference between the two frames of images, and conducts comparative experiments on the two methods using the correctly matched feature points as an indicator.

**Figure 7:**
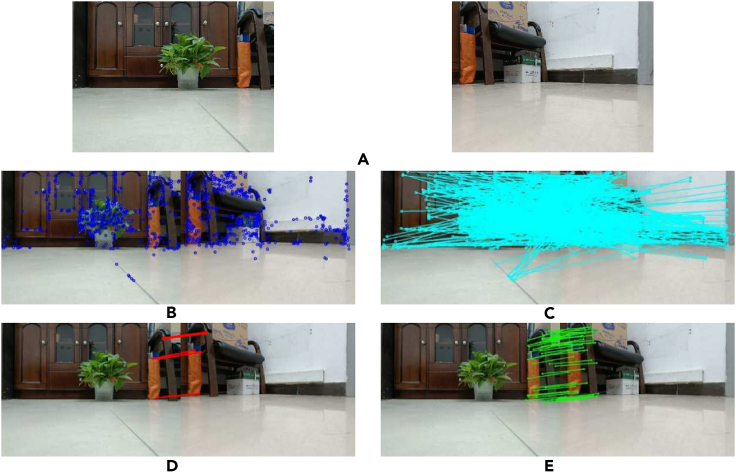
Experimental results of feature extraction and matching (A) image pair taken by robot rotating 80°; (B) feature point extraction results; (C) feature point pair matching; (D) correct feature point pairs after RANSAC elimination; (E) correct feature point pairs after BFS eliminated.

To better illustrate the effect, this article only gives a group of time continuous images with an angle difference of 80° in indoor scenes taken by mobile robots as an example to illustrate the effectiveness of the algorithm in this article, as shown in Figure 7.

Figure 7 shows the feature extraction and matching effects, respectively. It is not difficult to see that even after feature matching, there are still a large number of incorrect matching points, and if it is not processed, it is difficult to conduct subsequent pose prediction. Figure 7D shows the original RANSAC method, and Figure 7E shows the error-matching elimination effect of the BFS method. As shown in the figure, this algorithm improves the number of interior points and can effectively provide the correct number of feature points required for subsequent pose matrix calculations.

**Figure 8:**
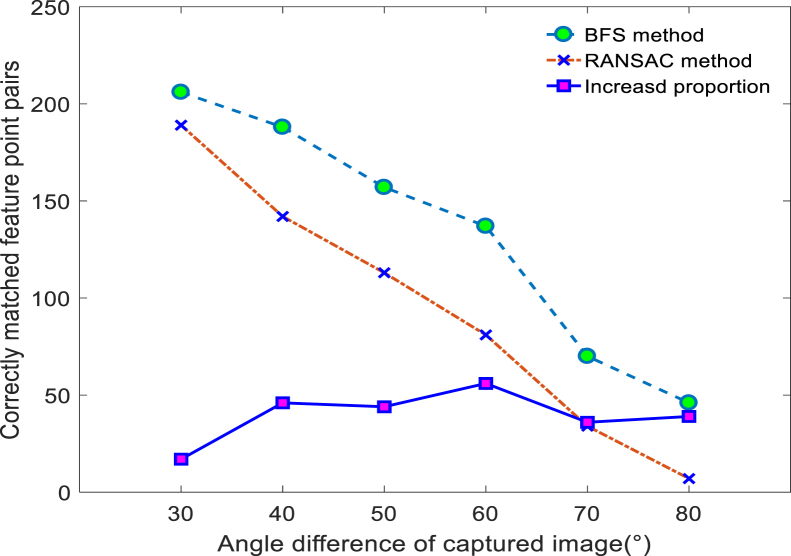
Correctly matched feature point comparison curve

Combining Figures 7 and 8, Table 1, it can be seen that within the range of 30°–80°, the correct feature point logarithms obtained by the BFS are always greater than those obtained by the RANSAC method. When angle rotation occurs, both methods can extract a large number of correct matching features when rotating by 30°, and as the angle gradually increases, the number of matching points extracted by both methods decreases to a certain extent. However, in the case of 80°rotation, the RANSAC method can only obtain 7 sets of correct matching points, while the BFS can obtain 46 sets of matching points. More correct matching point pairs can not only restore more 3D map points, but also ensure the robustness of system tracking.

**Figure 9:**
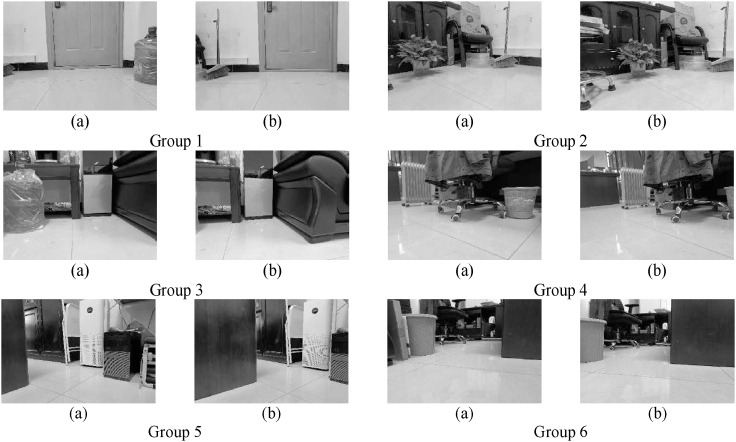
Image pairs taken by different robots (A) image taken by robot 1; (B) image taken by robot 2.

**Table 1:** Correctly matching feature points from different angles

Rotation angle	30°	40°	50°	60°	70°	80°
**BFS**	206	188	157	137	70	46
**RANSAC**	189	142	113	81	34	7
**Increased proportion**	17	46	44	56	36	39

### Similar scene identification experiment

In this section of the experiment, we first use the captured scene images as a training set to construct an ORB visual feature dictionary. In the process of training the dictionary, this article uses 31 images as input to the training set, sets each clustering parameter k = 10 and layer depth d = 6, and obtains a dictionary containing 15475 leaf nodes (words). Based on the key frame database method, the following six sets of images are constructed as a key frame database. In Figure 9, the image numbered (a) represents robot 1, and the image numbered (b) represents robot 2. Then, the similarity score is calculated to complete similar scene recognition.

The larger the data value in Table 2, the higher the pixel brightness in the grayscale image. It is not difficult to see that the element pixels at the diagonal position have the highest brightness, which means that each group of corresponding images has the highest similarity score, which is consistent with the human eye observation results. At the same time, the similarity score in different scenarios is relatively low, indicating that the design of constructing a key frame database and using it for similar scene recognition can effectively suppress scene mismatches. Using the method introduced in the previous chapter, the most similar scenes can be identified from images taken by different robots.

**Table 2:** Similarity score of images taken by different robots

Robot 2 Score Robot 1	First frame	Second frame	Three frame	Four frame	Five frame	Six frame
**First frame**	0.261	0.049	0.061	0.059	0.015	0.040
**Second frame**	0.096	0.299	0.073	0.068	0.010	0.088
**Three frame**	0.049	0.048	0.251	0.083	0.022	0.093
**Four frame**	0.028	0.061	0.053	0.236	0.017	0.093
**Five frame**	0.018	0.018	0.021	0.014	0.324	0.018
**Six frame**	0.054	0.075	0.051	0.113	0.010	0.365

### Single-robot verification experiment

For ease of explanation, this article names the single-robot SLAM system based on the bidirectional filtering strategy as SRV-SLAM, and the multi-robot SLAM system proposed using the method of constructing a key frame database and scene recognition as MRV-SLAM.

**Figure 10:**
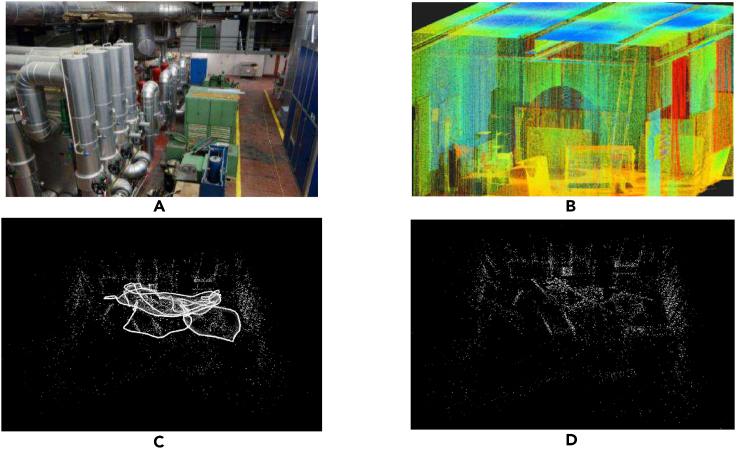
Single-robot exploration process under the EuRoC dataset V1-01 sequence (A) industrial plant; (B) workshop room; (C) movement trajectory; (D) point cloud map.

This section conducts experiments on the EuRoC dataset collected by the Federal Institute of Technology in Zurich to verify the effectiveness of the SRV-SLAM. This dataset was recorded using an MT9V034 camera, which can provide a synchronous binocular image of 20 Hz, and provide the real trajectory of the robot through a motion capture system, which can be effectively used for the evaluation of visual SLAM.As shown in Figures 10A and 10B. In order to verify the performance of the algorithm, we have done a lot of comparative experiments. To simplify the notation, we use BF-SLAM (the SLAM method based on the bidirectional filtering strategy) to represent our algorithm and compares it with the current state-of-the-art visual-inertial information fusion system VI-ORB-SLAM,^34^ ORB-SLAM3,^8^ RK-VIF-SLAM,^35^ and NeRF-SLAM.^36^

Absolute Pose Error (APE) is used to evaluate the accuracy of the positioning results of a single-robot SLAM system. The difference between the calculated posture and the actual posture, known as the APE value, can directly reflect the calculation accuracy and global consistency of the motion trajectory.

The system APE value at frame i is defined as follows:

(Equation 21) $$E_{i}=P_{i}^{-1}\cdot Q_{i}$$

Wherein, $P_{i}$ represents the true pose value of the dataset at time i, and $Q_{i}$ represents the estimated pose value of the SLAM system at time i. At this time, the root-mean-square error (RMSE) is used as the measurement index to calculate the overall trajectory error:

(Equation 22) $$RMSE\left(E_{1:N}\right)={\left(\frac{1}{N}\sum_{i=1}^{N}{\left\|trans\left(E_{i}\right)\right\|}^{2}\right)}^{1/2}$$

Where *N* represents the total number of keyframes on the entire trajectory.

Figure 10 shows the exploration process of BF-SLAM under the EuRoC dataset V1_01 sequence. Figure 10C shows the motion trajectory of the robot, which is connected by the position and posture of the key frames at each moment. From the figure, it is not difficult to see that due to the similarity of captured images in some scenes during the operation of the robot, the motion trajectory forms a closed loop, and the overall positioning accuracy of the system is high; Figure 10D shows a robot modeling representation of the exploration environment, showing a rough outline of the robot exploration scene.

**Figure 11:**
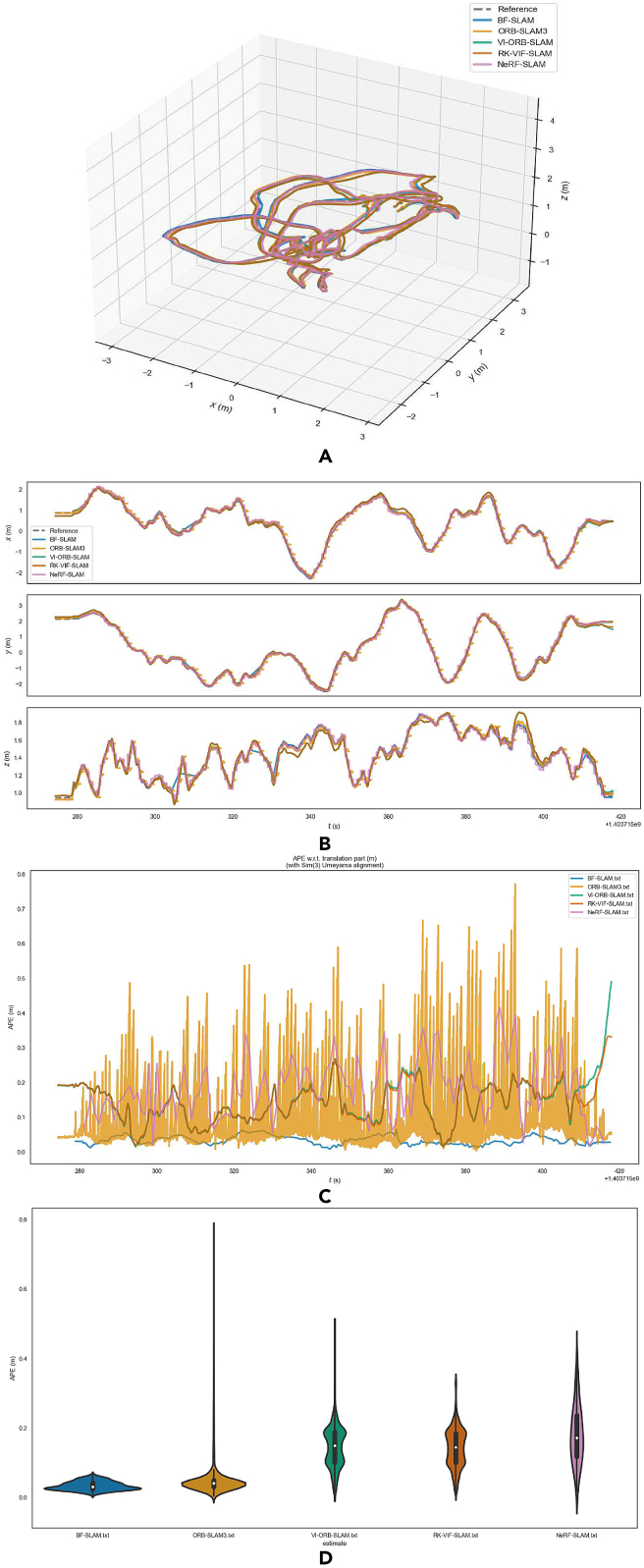
Comparison chart of algorithm errors under EuRoC dataset V1-01 sequence (A) Trajectory; (B) Track accuracy; (C) Comparison of APE values; (D) APE statistical property.

Figure 11 shows the algorithm error comparison between BF-SLAM, ORB-SLAM3, VI-ORB-SLAM, RK-VIF-SLAM, and NeRF-SLAM under the V1-01 sequence. Combining Table 3, it is effectively illustrated that the BF-SLAM proposed in this article has a higher positioning accuracy than the original ORB-SLAM3, VI-ORB-SLAM, RK-VIF-SLAM, and NeRF-SLAM under the condition that APE is taken as the measurement index. Compared with the original algorithm, the improved statistical indicators have been reduced to a certain extent, which proves the effectiveness of this improvement.

**Table 3:** Statistical characteristics table of APE under EuRoC dataset V1-01 sequence (unit: m)

	RMSE	Average value	Median value	Minimum value	Standard deviation
**BF-SLAM**	0.036	0.034	0.031	0.009	**0.013**
**ORB-SLAM3**	0.087	0.056	0.041	0.004	0.067
**VI-ORB-SLAM**	0.159	0.147	0.150	0.015	0.062
**RK-VIF-SLAM**	0.154	0.143	0.145	0.016	0.056
**NeRF-SLAM**	0.146	0.122	0.103	0.007	0.080

### Multi-robot verification experiment

In order to more intuitively reflect the advantages of multi-robot collaboration, this article compares M-BFSI-SLAM (the multi-robot SLAM method based on bidirectional filtering and scene identification), SRV-SLAM (the single-robots visual SLAM method) and VI-ORB-SLAM, ORB-SLAM3, RK-VIF-SLAM, and NeRF-SLAM scene splicing in the above design scenario. The comparison content includes the following three criteria: first, compare the positioning accuracy of the two schemes; Secondly, compare the efficiency of the two schemes in mapping the same environment; Third, compare the map representations of the two schemes for the same environment.

**Figure 12:**
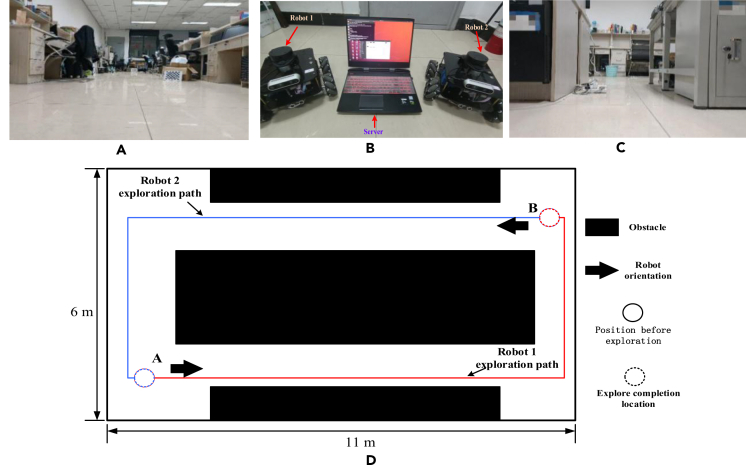
Experimental conditions for the M-BFSI-SLAM system (A) indoor experiment scene A; (B) hardware platform; (C) indoor experiment scene B; (D) design of sub-robot travel path.

**Figure 13:**
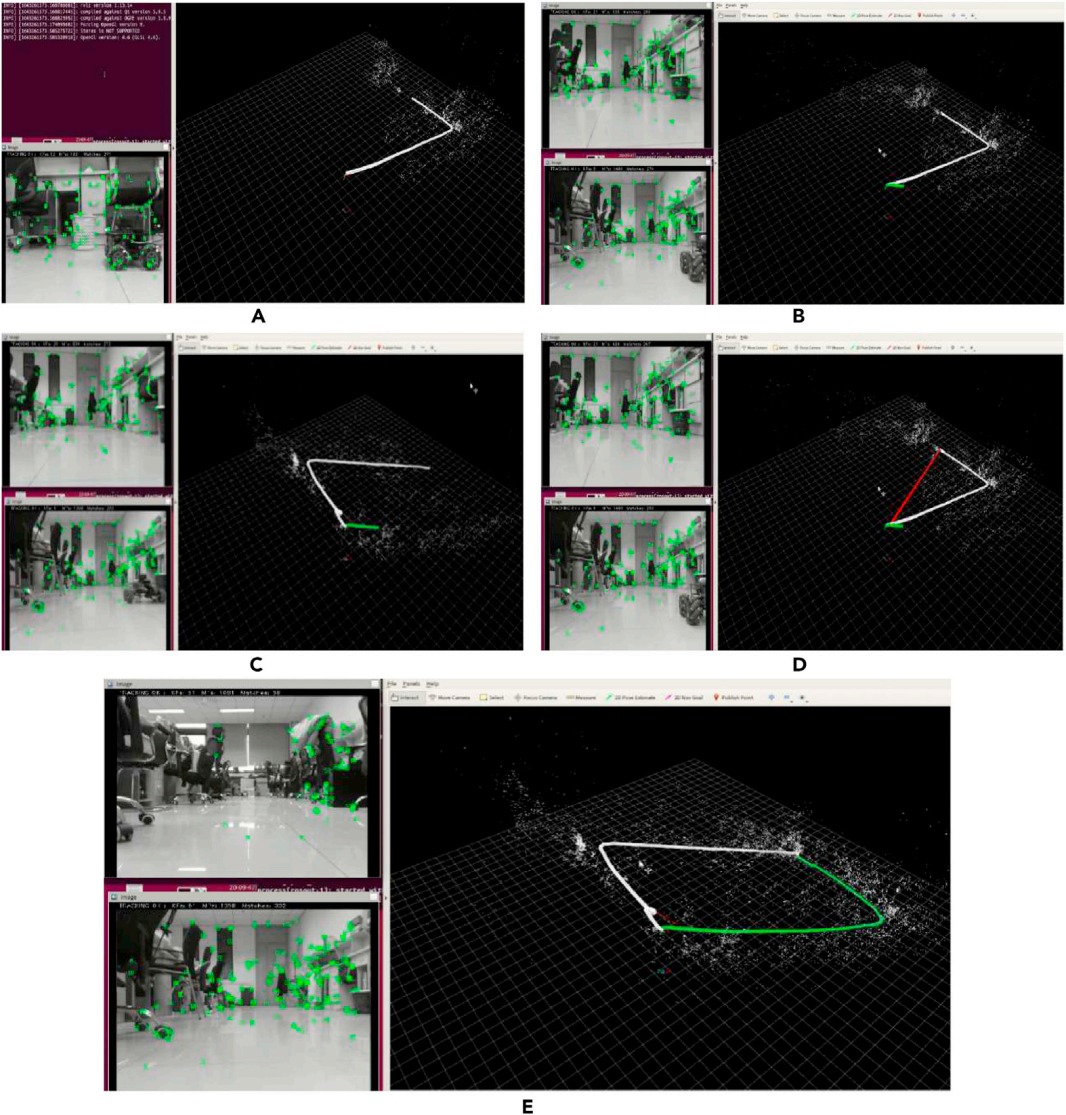
M-BFSI-SLAM operation results (A) schematic diagram of robot 1 exploration; (B) schematic diagram of robot 2 exploration; (C)similar scene identification; (D) coordinate system conversion; (E) operation results after coordinate system conversion.

First, compare the positioning accuracy of the two schemes. Place the two robots in their starting positions according to the design in Figure 12D. The robot 1 exploration process is shown in Figure 13. The white trajectory is the SLAM system’s position estimation of itself, and the white point set is the point cloud map generated during the robot exploration process. When robot 1 turns left, robot 2 is observed in the field of vision.

As shown in Figure 13A, the exploration of robot 1 is completed, and its visual angle orientation is the same as that of robot 2 to be launched. In Figure 13B, the lower left view shows the image returned by Robot 1 to the laptop, while the upper left view shows the image returned by Robot 2 to the laptop. At this point, Robot 2 starts out, as shown in Figure 13C. During the journey, the host computer uses the incrementally constructed key frame database and the key frames returned by Robot 2 to detect that the exploration endpoint of Robot 1 is similar to the exploration start point of Robot 2, match the key frames, and solve the transformation matrix. Finally, the local map of robot 2 was successfully transferred to the coordinate system of robot 1 through the transformation matrix. As can be seen from Figure 13D, the end position of robot 1 basically coincides with the initial position of robot 2, which proves that the multi-robot collaborative SLAM scheme designed in this article achieves effective closed-loop robot trajectory while efficiently exploring the environment.

**Figure 14:**
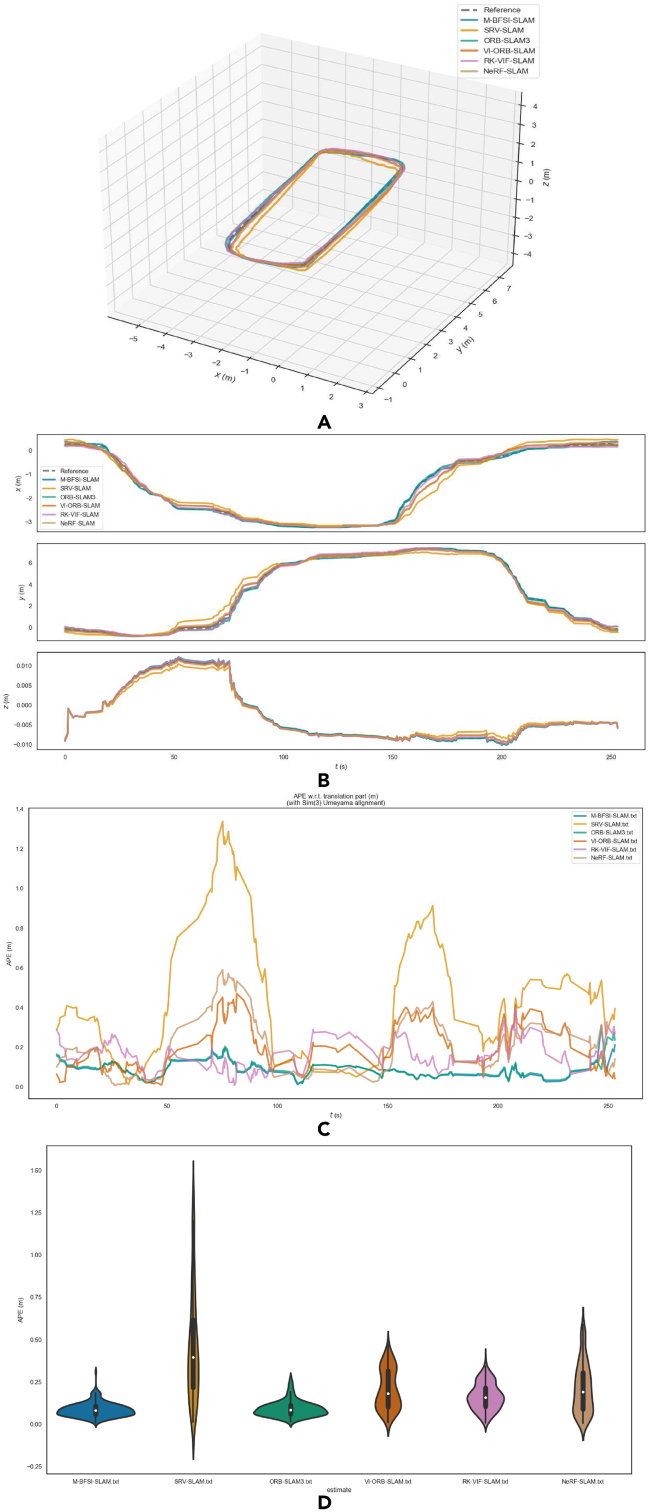
Experimental results in complex indoor scenes (A) 3D trajectory in the indoor scene; (B) track accuracy curve in indoor scenes; (C) comparison of APE values; (D) APE statistical property.

The green trajectory in Figure 13E shows the motion trajectory of robot 2. After completing the single-robot scheme and multi-robot scheme experiments, draw their motion trajectory diagrams shown in Figure 14A.

The total length of the reference trajectory is 20.969 m, the estimated trajectory length of M-BFSI-SLAM is 20.988m, and the estimated trajectory length of SRV-SLAM is 25.421m, ORB-SLAM3 is 21.012m, VI-ORB-SLAM is 21.145m, RK-VIF-SLAM is 20.818m, NeRF-SLAM is 20.772m. As can be seen from Figure 14B, compared to the estimated trajectory of SRV-SLAM, the estimated trajectory of the multi-robot SLAM system is more accurate and more closely aligned with theoretical values. The reason for this is that a single robot inevitably generates cumulative errors during the pose estimation process, which increase with the increase of robot exploration time and distance. Even if loop back detection reduces the cumulative error, the effect is still not ideal. In multi-robot system, exploration tasks for the entire environment are assigned to multiple sub-robots, with a relatively small amount of exploration tasks for each sub-robot. The SLAM process runs in a short time, and its cumulative error is smaller than that of a single-robot exploration scheme.

Analogous to the quantitative analysis method of the single-robot SLAM scheme in Section simulation experiment and result analysis, this section still uses APE as a quantitative indicator for error analysis. Figure 14 shows the comparison of exploration errors between SRV-SLAM, ORB-SLAM3, VI-ORB-SLAM, RK-VIF-SLAM, NeRF-SLAM and M-BFSI-SLAM in indoor environments. Combining Figure 14 and Table 4, it is effectively illustrated that the multi-robot collaborative SLAM scheme M-BFSI-SLAM proposed in this article has a higher positioning accuracy than the single-robot scheme VI-ORB-SLAM, ORB-SLAM3, RK-VIF-SLAM, NeRF-SLAM and SRV-SLAM under the condition that APE is taken as the measurement index. At the same time, after expanding to the multi-robot collaboration scheme, various statistical indicators have been reduced to a certain extent, indicating that the multi-robot collaboration SLAM proposed in this article can effectively reduce cumulative errors and obtain more accurate exploration trajectory by reducing the running time of sub-robot SLAM through multi-machine task allocation.

**Table 4:** Statistical characteristics of APE in complex indoor scenes (unit: m)

	RMSE	Average value	Median value	Minimum value	Standard deviation
**M-BFSI-SLAM**	0.096	0.087	0.823	0.111	0.041
**SRV-SLAM**	0.567	0.465	0.395	0.13	0.325
**ORB-SLAM3**	0.104	0.093	0.085	0.017	0.046
**VI-ORB-SLAM**	0.237	0.206	0.183	0.010	0.118
**RK-VIF-SLAM**	0.182	0.165	0.159	0.008	0.078
**NeRF-SLAM**	0.262	0.216	0.192	0.006	0.148

Secondly, compare the efficiency of the two schemes in mapping the same environment. The efficiency of the two schemes is expressed by counting their mapping time. To unify the measurement standards, when controlling the movement of mobile robots, the linear speed is set to the minimum value of 0.1 m/s, and there is no pause during the movement. The comparison of mapping time is shown in Table 5.

**Table 5:** Comparison of drawing time in indoor environment (uint: s)

Scheme	Exploration time	
**M-BFSI-SLAM**	Robot 1	**216**
	Robot 2	**229**
**SRV-SLAM**	Robot 1	**269**
	Robot 2	**281**
**ORB-SLAM3**	Robot 1	**259**
	Robot 2	**262**
**VI-ORB-SLAM**	Robot 1	**266**
	Robot 2	**269**
**RK-VIF-SLAM**	Robot 1	**258**
	Robot 2	**263**
**NeRF-SLAM**	Robot 1	**285**
	Robot 2	**290**

As can be seen from the Table 5, compared to the single-robot scheme, the travel path length of each sub-robot in a multi-robot system is about 1/2 of its length, and the exploration time is shorter. When two robots start and move forward at the same time, the total time required for local mapping should be 229s. If Robot 1 first reaches the predetermined position, a similar scene is observed at B in Figure 12D, and scene identification is performed at this time. After repeated experiments, the time required for map fusion in this indoor scene is around 4s, calculated as 4s. Finally, the total time spent on drawing for multi-robot scheme should be 229 + 4 = 233s, which is far less than for a single-robot scheme. If you are in a larger scene, key frame search and matching consumes more computational resources, and map fusion time will increase to a certain extent. However, with larger scenes, more robots are often invested in collaboration, which inevitably further increases the efficiency of mapping, reducing the negative impact of time-consuming map fusion, making large-scale mapping possible.

**Figure 15:**
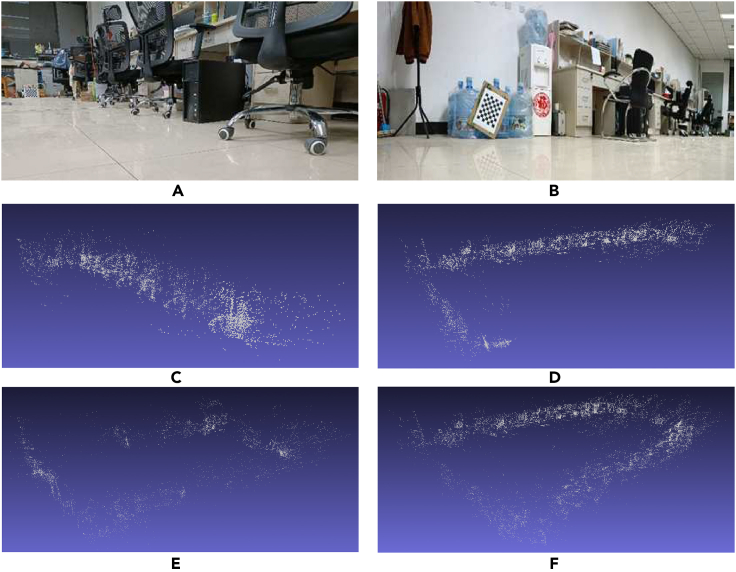
Multi-robot solution point cloud map (A) robot 1 explore Scene; (B) robot 2 explore Scene; (C) robot 1 point cloud map; (d) robot 2 point cloud map; (E) SRV-SLAM point cloud map; (F) M-BFSI-SLAM point cloud map.

Third, compare the map representations of the two schemes for the same environment. Display the rendering effect of a single robot, the rendering effect of sub-robot 1, the rendering effect of sub-robot 2, and the rendering effect of multi-robot splicing, as shown in Figure 15.

As can be seen from Figure 15, for the multi-robot scheme, the local map of each sub-robot can reproduce the environment contour to a certain extent. When faced with similar scenes during exploration, similar key frames are quickly identified and conversion matrices are calculated, thereby completing the stitching of submaps. Compared to the exploration time of a single-robot scheme, even if the multi-robot scheme sacrifices a certain exploration time for more dense mapping effects, the total system operation time is still less than that of a single-robot system. The final mosaic of the global map not only has good consistency, but also has a denser rendering effect. Table 6 compares the map representation efficiency of the two schemes for the same environment by counting the number of key frames and map points. As mentioned earlier, while exploring with less time, the multi-robot scheme provides a more intuitive description of the map.

**Table 6:** Map representation efficiency in the same environment

	Partial Map 1	Partial Map 2	SRV-SLAM map	M-BFSI-SLAM map
Number of keyframes	128	162	237	290
Number of map points	10131	12648	7992	20877

### Conclusions

Aiming at the problem that ORB-SLAM faces when rotating at a large angle, which leads to the incorrect matching of features, leading to tracking failures, this article introduces a bidirectional filtering mechanism and proposes an improved RANSAC error-matching elimination algorithm. This algorithm not only effectively retains the original correct matching feature pairs, but also reduces the instability of model iterations and model failures caused by input data. Experiments show that the proposed algorithm can effectively provide the correct matching point pairs required for pose resolution, improve robot motion trajectory estimation, and establish a good foundation for multi-robot collaborative SLAM. Then, inspired by closed-loop detection, a method of constructing a key frame database for detecting similar scenes between sub-robots is proposed to solve the problem of map mosaic between machines. A key frame database is constructed in the upper computer, and for the two best-matched frames, the preliminary calculation of the pose conversion matrix is completed through matching point pairs. Then, using all the best-matched feature point pairs, the global optimization is performed using the pose matrix as the initial value to obtain an optimal transformation matrix. After completing the global map mosaic, due to the relativity of the reference coordinate system at the transformation time, the transformed point cloud is converted to the initial motion time of the robot, and thus the multi-robot collaborative SLAM is completed. Finally, the multi-robot collaborative SLAM scheme designed in this article is validated through indoor complex scene experiments. Compared with the single-robot SLAM scheme in terms of positioning accuracy, mapping efficiency, and map representation, the multi-robot environment modeling method based on bidirectional filtering and scene recognition proposed in this article not only has higher positioning accuracy, but also has richer map representations, providing a feasible solution for large-scale scene exploration.

## STAR★Methods

### Key resources table

**Table:** 

REAGENT or RESOURCE	SOURCE	IDENTIFIER
**Deposited data**		

Experimental dataset	EuRoC	https://projects.asl.ethz.ch/datasets
Theoretical results	This paper	Section “conclusions”

**Software and algorithms**		

Ubuntu	Linux	https://www.linux.org/
Python3.7	Python Software Foundation	https://www.python.org/
VI-ORB-SLAM	Mur-Artal et al.^3^^,^^34^	https://doi.org/10.1109/LRA.2017.2653359
ORB-SLAM3	C Campos et al.^8^	https://doi.org/10.1109/TRO.2021.3075644
NeRF-SLAM	CVPR	https://www.thecvf.com/
RK-VIF-SLAM	Cui et al.^35^	https://doi.org/10.1109/LRA.2017.2653359

### Resource availability

#### Lead contact

Further information and requests for resources should be directed to the lead contact, Jiashan Cui, Xidian University School of Aerospace Science and Technology, No.266 Xinglong Section, Xifeng Road, Xi’an City, Shaanxi Province, Xi’an 710126, China. Email: jscui@xidian.edu.cn.

#### Materials availability

The source of materials is displayed in the key resources table

#### Data and code availability

Data:
  Data S1: single robot experiments, related to Figs 7, 8, 9, and 10.
  Data S2: multi robot experiments, related to Figs. 11, 12, 13, 14, and 15.
  The dataset generated during this study is available at https://github.com/huazhaowz/MBF_SLAM.
Code: The codes used for data analyses are available at https://github.com/huazhaowz/MBF_SLAM.
Any additional information required to reproduce the study and reanalyze the data reported in this paper is available from the lead contact upon request.

### Method details

All the method details are described in the main text (See “map splicing strategy” section).

#### Quantification and statistical analysis

All the statistical analysis and the results are described in the main text (See “simulation experiment and result analysis” section). Data are displayed in Figures 5, 7, 9, 10, 11, 13, and 15.
