# Supplementary figures and images for: A fast and high precision multi-robot environment modeling based on M-BFSI: Bidirectional filtering and scene identification method
Source: iScience. 2024 Apr 10;27(5):109721. doi: 10.1016/j.isci.2024.109721 (PMC11068629; doi:10.1016/j.isci.2024.109721)

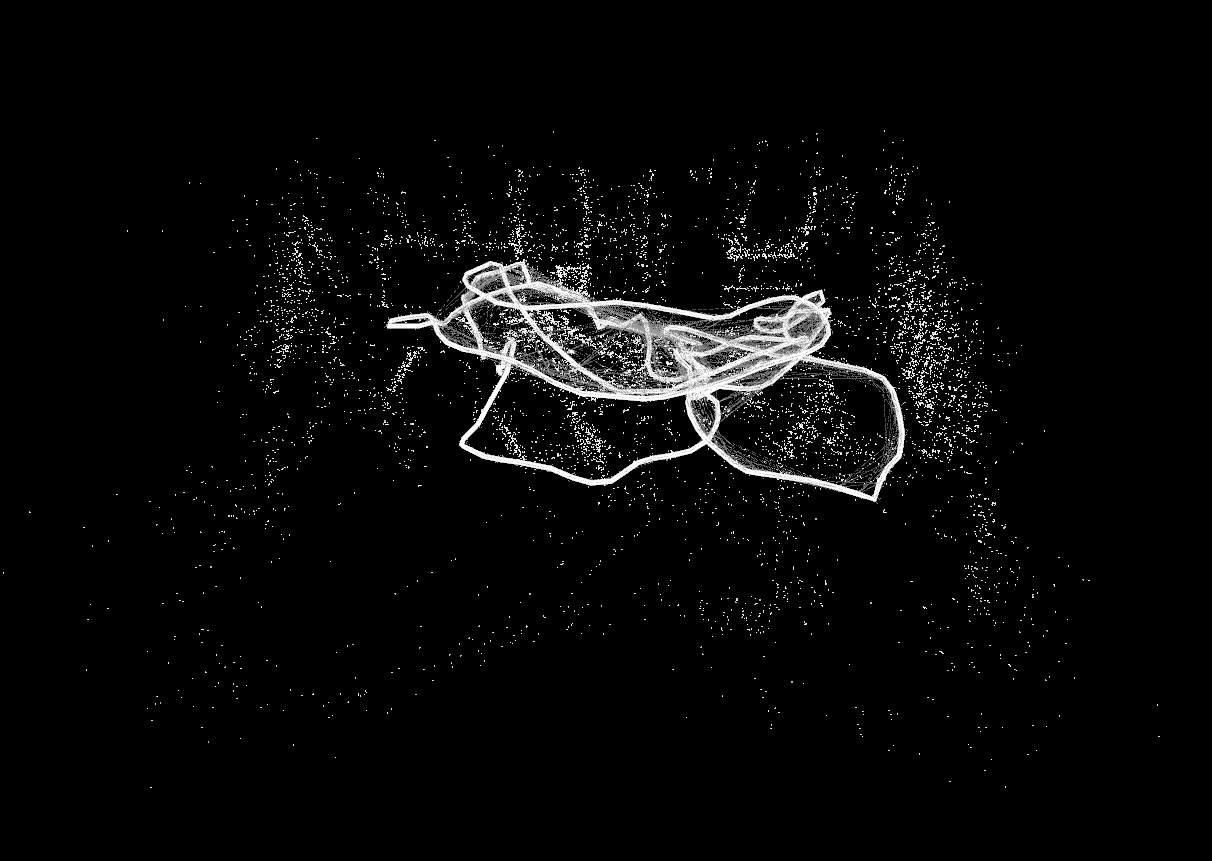

Supplement: Document S1. Data S1 and S2 [file mmc1.zip › Data/Data S1:single-robot,related to Figure 7,10,11/Data S1-2:pointcloud of single-robot,related to Figure 10/pointcloud1.png]

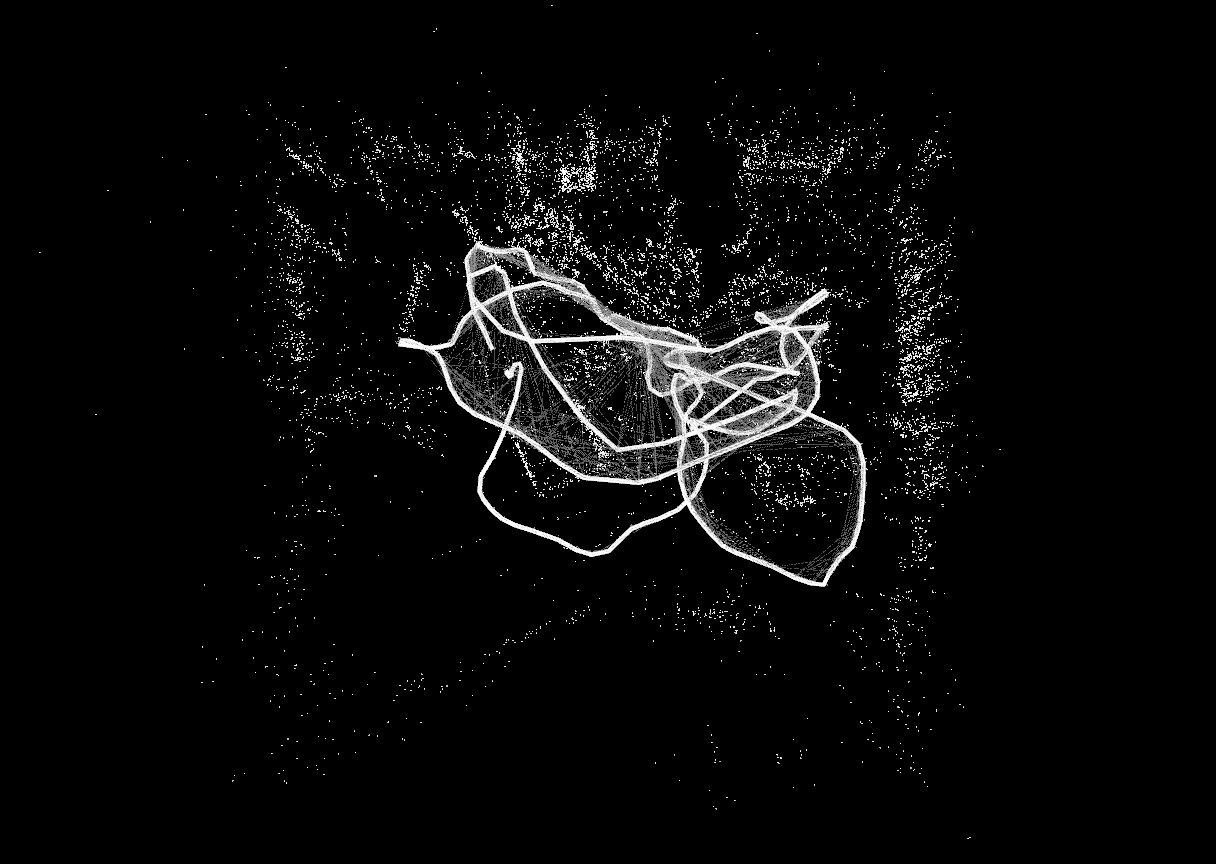

Supplement: Document S1. Data S1 and S2 [file mmc1.zip › Data/Data S1:single-robot,related to Figure 7,10,11/Data S1-2:pointcloud of single-robot,related to Figure 10/pointcloud2.png]

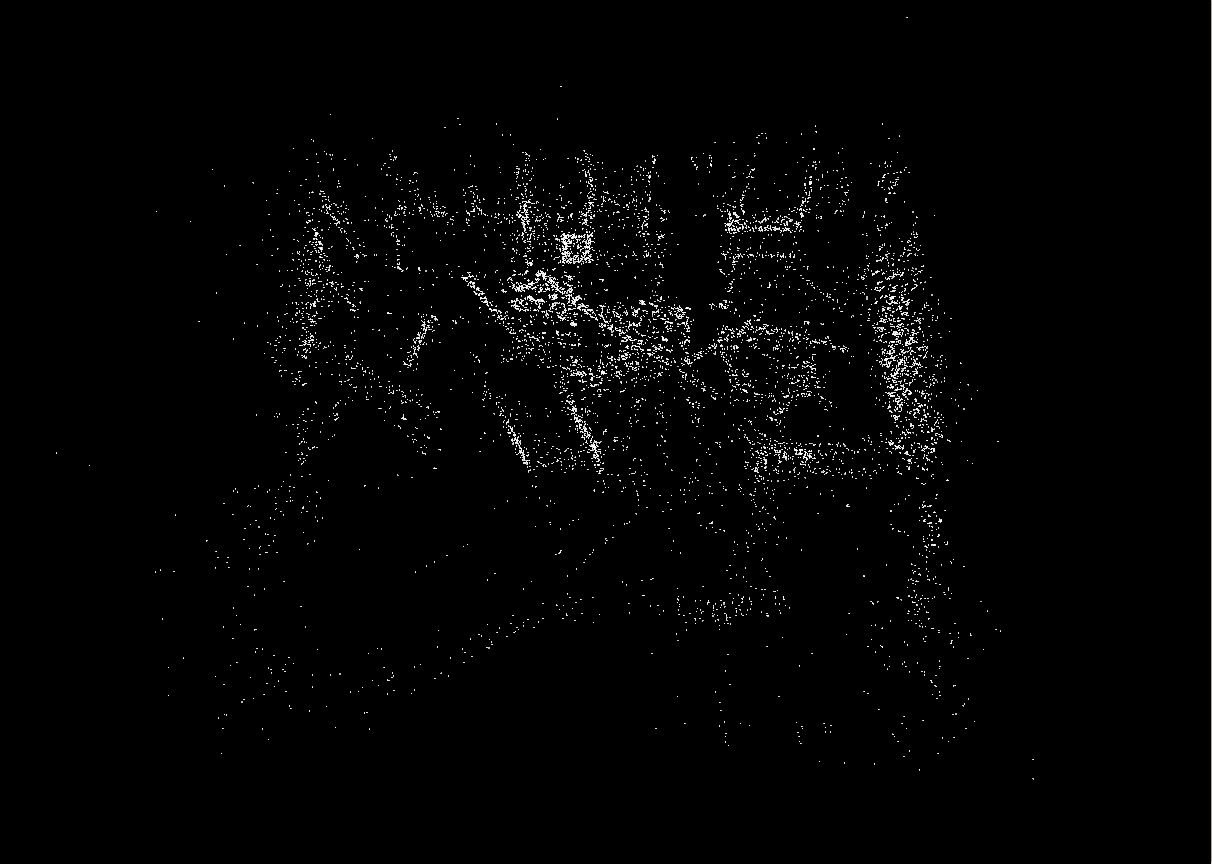

Supplement: Document S1. Data S1 and S2 [file mmc1.zip › Data/Data S1:single-robot,related to Figure 7,10,11/Data S1-2:pointcloud of single-robot,related to Figure 10/pointcloud3.png]

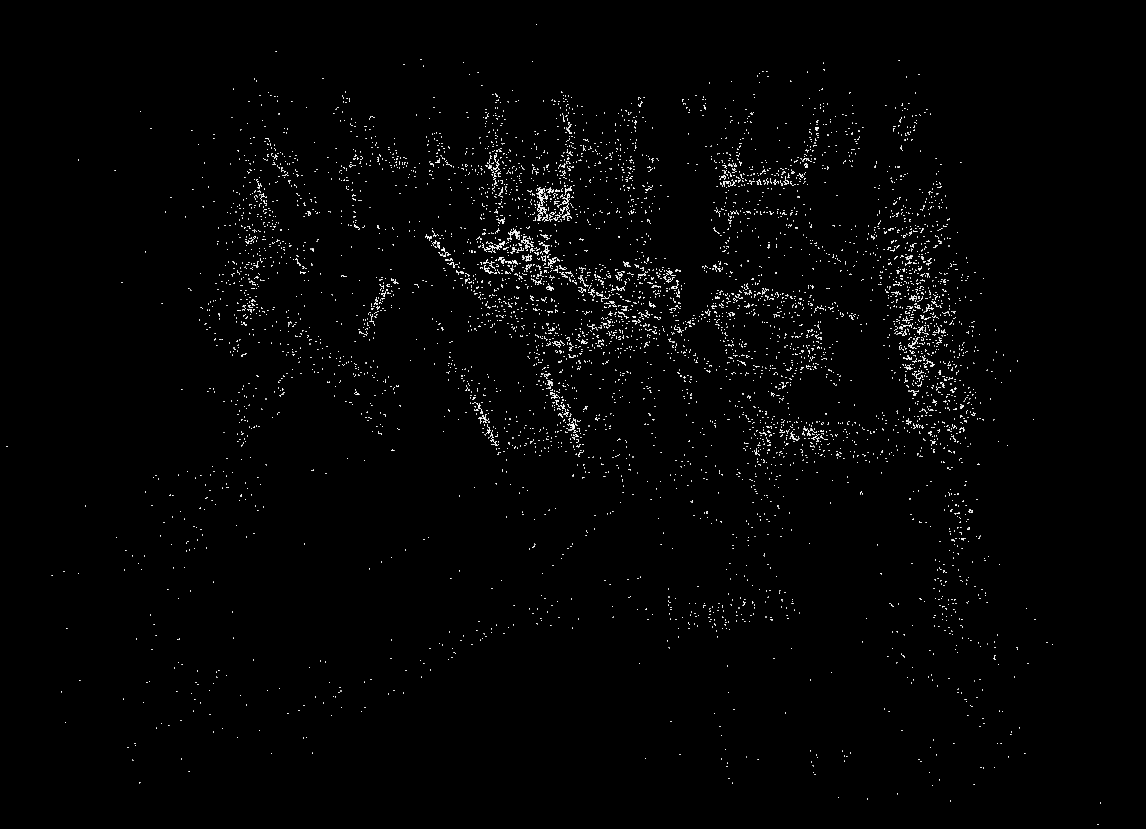

Supplement: Document S1. Data S1 and S2 [file mmc1.zip › Data/Data S1:single-robot,related to Figure 7,10,11/Data S1-2:pointcloud of single-robot,related to Figure 10/pointcloud4.png]

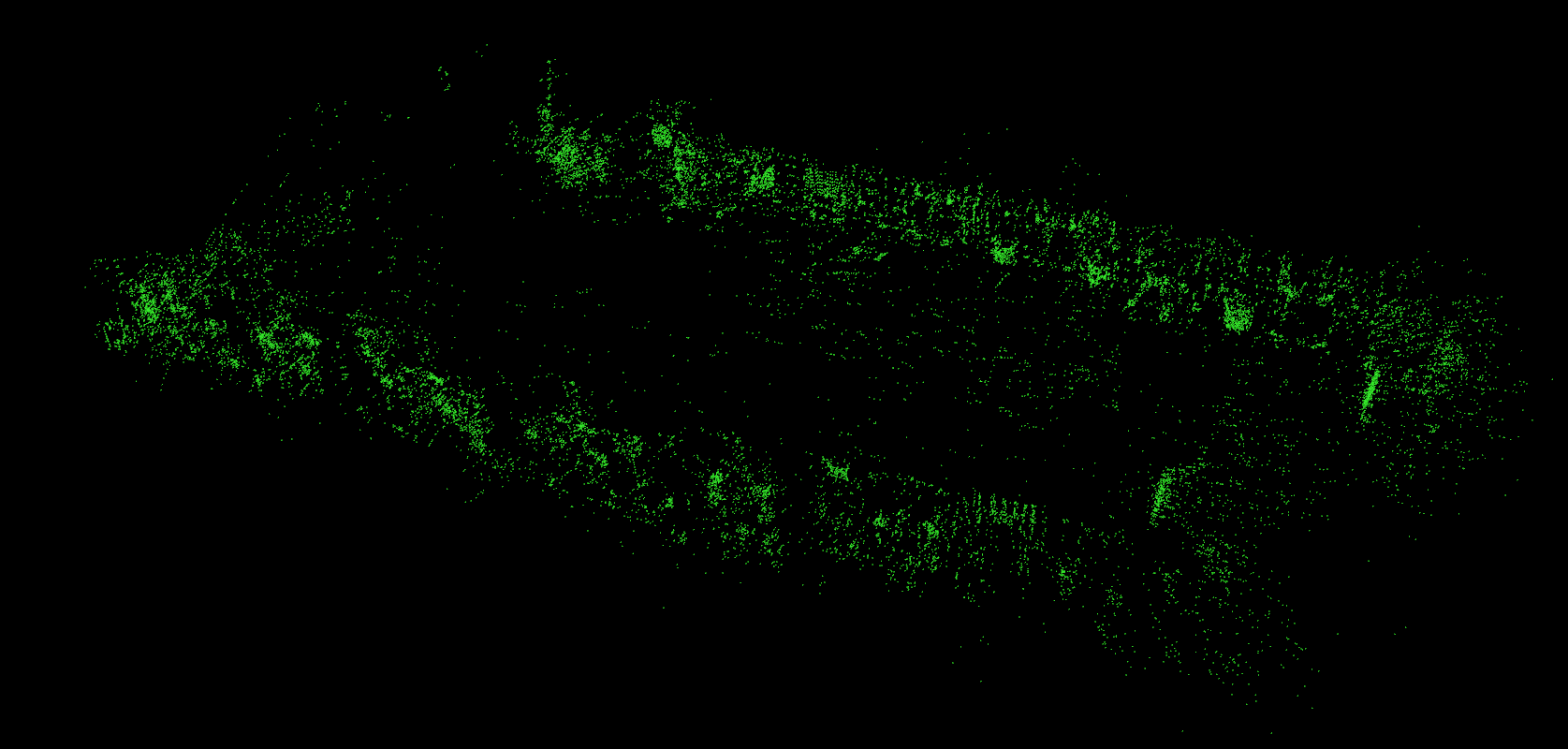

Supplement: Document S1. Data S1 and S2 [file mmc1.zip › Data/Data S2:multi-robots,related to Figure13-15/Data S2-1:point cloud map, related to Figure15/merged_result1.png]

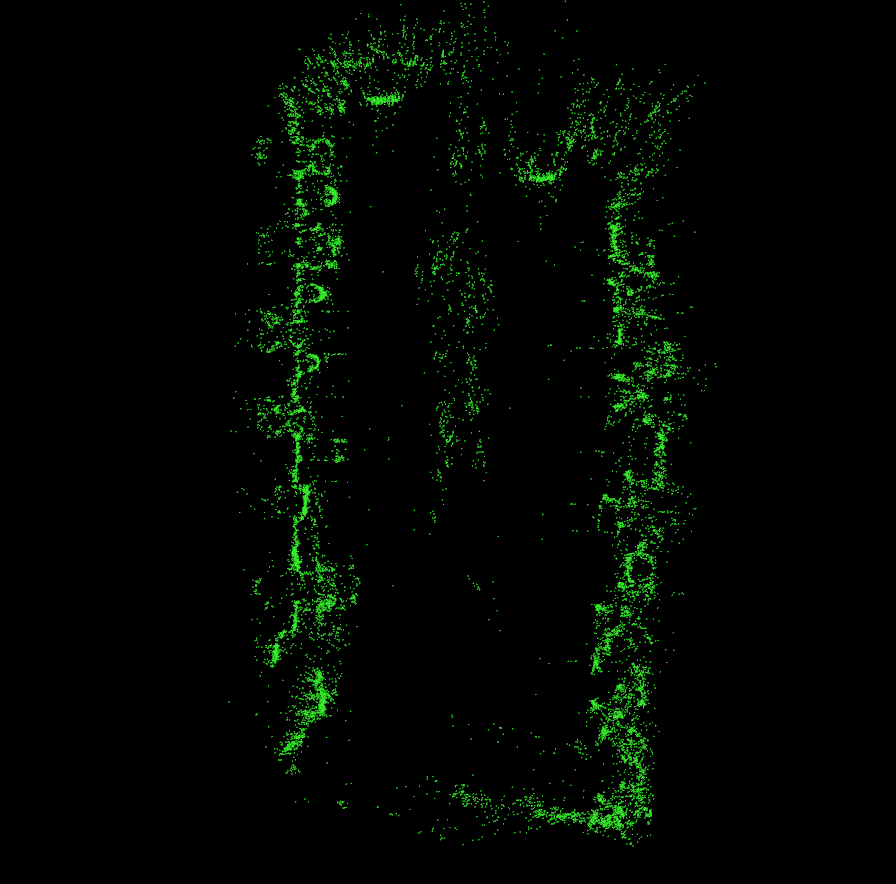

Supplement: Document S1. Data S1 and S2 [file mmc1.zip › Data/Data S2:multi-robots,related to Figure13-15/Data S2-1:point cloud map, related to Figure15/merged_result2.png]

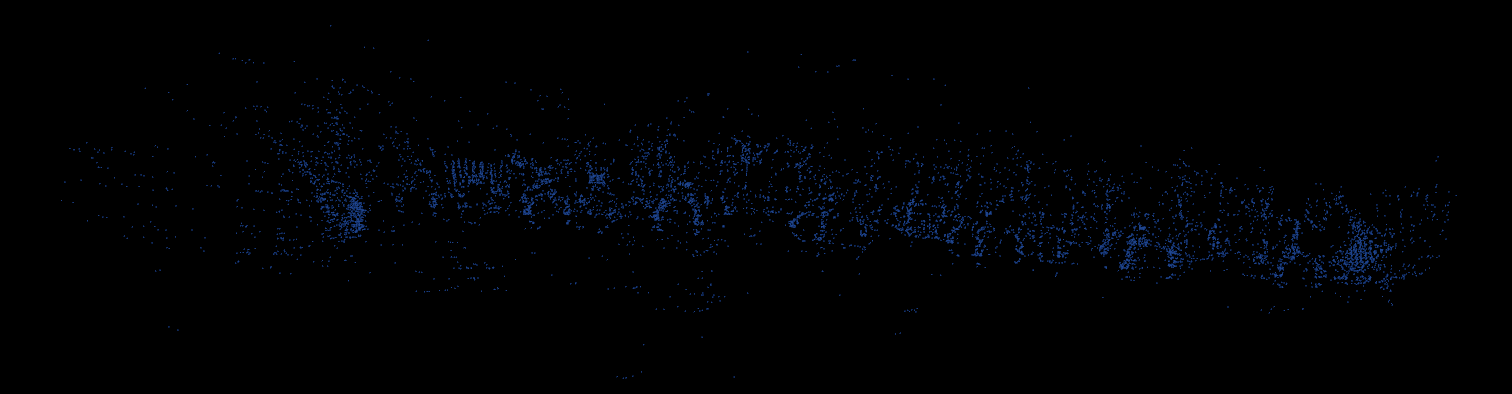

Supplement: Document S1. Data S1 and S2 [file mmc1.zip › Data/Data S2:multi-robots,related to Figure13-15/Data S2-1:point cloud map, related to Figure15/robot1.png]

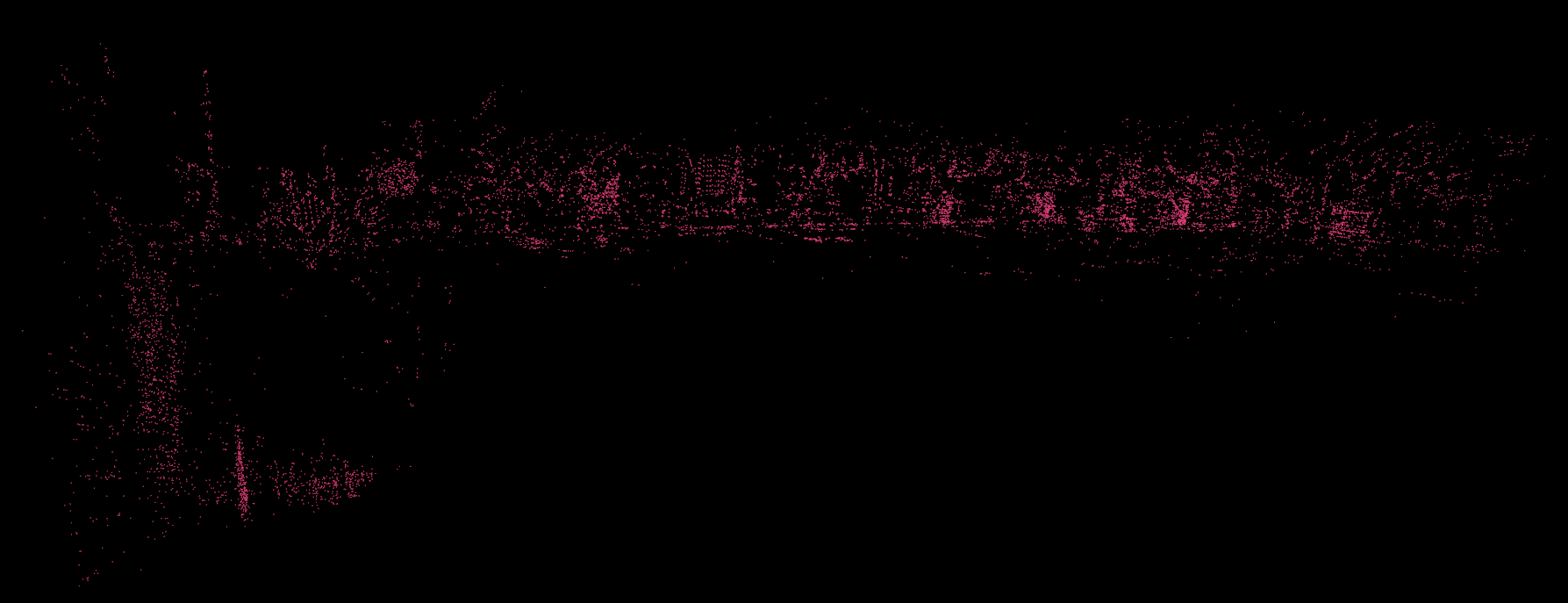

Supplement: Document S1. Data S1 and S2 [file mmc1.zip › Data/Data S2:multi-robots,related to Figure13-15/Data S2-1:point cloud map, related to Figure15/robot2.png]
